# Robots engage face-processing less strongly than humans

**DOI:** 10.3389/fnrgo.2022.959578

**Published:** 2022-10-20

**Authors:** Ali Momen, Kurt Hugenberg, Eva Wiese

**Affiliations:** ^1^Warfighter Effectiveness Research Center, United States Air Force Academy, Colorado Springs, CO, United States; ^2^Department of Psychology, George Mason University, Fairfax, VA, United States; ^3^Department of Psychological and Brain Sciences, Indiana University, Bloomington, IN, United States; ^4^Institute for Psychology and Ergonomics, Technical University of Berlin, Berlin, Germany

**Keywords:** human–robot interaction, human–agent interaction, social cognition, face-processing, anthropomorphism

## Abstract

Robot faces often differ from human faces in terms of their facial features (e.g., lack of eyebrows) and spatial relationships between these features (e.g., disproportionately large eyes), which can influence the degree to which social brain [i.e., Fusiform Face Area (FFA), Superior Temporal Sulcus (STS); Haxby et al., 2000] areas process them as social individuals that can be discriminated from other agents in terms of their perceptual features and person attributes. Of interest in this work is whether robot stimuli are processed in a less social manner than human stimuli. If true, this could undermine human–robot interactions (HRIs) because human partners could potentially fail to perceive robots as individual agents with unique features and capabilities—a phenomenon known as outgroup homogeneity—potentially leading to miscalibration of trust and errors in allocation of task responsibilities. In this experiment, we use the face inversion paradigm (as a proxy for neural activation in social brain areas) to examine whether face processing differs between human and robot face stimuli: if robot faces are perceived as less face-like than human-faces, the difference in recognition performance for faces presented upright compared to upside down (i.e., inversion effect) should be less pronounced for robot faces than human faces. The results demonstrate a reduced face inversion effect with robot vs. human faces, supporting the hypothesis that robot faces are processed in a less face-like manner. This suggests that roboticists should attend carefully to the design of robot faces and evaluate them based on their ability to engage face-typical processes. Specific design recommendations on how to accomplish this goal are provided in the discussion.

## Introduction

When interacting with groups of non-human entities, such as robots or avatars, we tend to perceive them as homogenous groups of agents that all have similar characteristics and capabilities (Keller and Rice, 2009). This overgeneralization of features from one agent to another is often due to a lack of familiarity with or a lack of motivation to process non-social agents, leading to a reduction in brain areas [i.e., Fusiform Face Area (FFA), Superior Temporal Sulcus (STS)] specialized to perceive agents as having identities with unique personality attributes and characteristics that be utilized to discriminate between them. Social agents, however, engage brain areas that process stimuli at the individual level (Hugenberg et al., 2010). In human–robot teams, this failure to perceive robot team members as individual social agents with unique characteristics and abilities can undermine trust and performance, such that knowledge about one robot could be erroneously overgeneralized to other robot team members (Geels-Blair et al., 2013). The consequence is that trust in robot team members is either too high (positivity bias, if initial experience with robots was positive) or too low (negativity bias, if initial experience was negative), resulting in overreliance in the former case, and distrust in the latter case (Parasuraman and Riley, 1997). The belief that all agents in a system are interchangeable leads to System-Wide Trust—an omnibus belief in the overall trustworthiness of the system—which undermines team performance when relying on robots that perform below expectations, and an increase in workload of the human operator when distrusting robots that perform above expectations (Parasuraman and Riley, 1997; Geels-Blair et al., 2013). A better calibration of trust could be achieved if human operators perceived robot team members as identifiable social entities with unique characteristics and abilities rather than a homogenous group of agents with similar features and capabilities. This phenomenon, known as Component-Specific Trust, is associated with improved performance and attitudes in human–robot teams and is established when human operators develop the ability to successfully differentiate specific agents within a system leading to better-calibrated, agent-specific beliefs (Keller and Rice, 2009; Geels-Blair et al., 2013).

But to generate component-specific trust, one must be able to perceive a robot agent as a social entity at the individual level to distinguish between them. Fortunately, extensive research has examined the phenomenon of seeing agents as unique social entities with identifiable personality attributes vs. interchangeable. Indeed, a long established phenomenon in perception is the tendency to see non-social stimuli as more similar than they are—a phenomenon known as outgroup homogeneity (Quattrone and Jones, 1981; Chance and Goldstein, 1996; Hugenberg et al., 2010). Outgroup homogeneity causes people to perceive agents as having features that blend in with others rather than individuating features that distinguish the agent from others (Hugenberg et al., 2010). Perception of others as unique social individuals rather than homogenous depends on two factors—familiarity with a class of stimuli and motivation (i.e., social relevance of agent/group/situation to self)—and in turn determines how difficult it is to perceive differences between individual agents (Fiske and Neuberg, 1990). Outgroup homogeneity occurs with little cognitive effort, does not require familiarity with the stimulus, and consumes so few cognitive resources that categorically relevant information can be extracted under high task-load or when being irrelevant to the task (Looser and Wheatley, 2010; Schein and Gray, 2015; Martini et al., 2016; Mandell et al., 2017; Wiese et al., 2018) whereas processing an agent as a social entity on the individual level engages the extraction of identifiable information from the stimuli, which requires more time and effort, and only happens when perceivers are sufficiently familiar with the stimuli and/or motivated (i.e., consider stimuli as part of their ingroup) to discriminate between stimuli (Fiske and Neuberg, 1990; Cloutier et al., 2005; Macrae et al., 2005).

The most important cues for perceiving an agent as social individuals with unique personality attributes in human–human interaction are derived from the face region: faces encode information relevant to identity, such as gender, age, or ethnicity, personality attributes, and characteristics as well as information regarding internal states, such as emotions and intentions (Haxby et al., 2000), and human brain areas sensitive to processing social stimuli during interactions are specifically implicated in the processing of human faces. For example, the FFA processes characteristics of faces that are unchangeable, such as an individual's identity. The STS, on the other hand, processes changeable characteristics of faces, such as an individual's internal states.

In line with this neural specialization, behavioral data suggests that faces are processed in a unique manner, relative to most other stimuli: whereas most visual stimuli are processed via a piecemeal integration of their separate features, faces are typically processed configurally, as an integrated Gestalt (see Maurer et al., 2002; for a review). This configural processing style leads to a variety of unique effects (Deska and Hugenberg, 2017), and causes perceivers to be sensitive to the orientation in which human faces are presented. When faces are presented upright, perceivers can process faces configurally, supporting strong face encoding and recognition. When faces are presented upside-down, however, configural processing is disrupted and participants are significantly less capable of recognizing familiar faces. Most importantly, this inversion effect does not occur for non-face stimuli (e.g., cars/dogs; Diamond and Carey, 1986; Rossion and Curran, 2010) and emphasizes the uniqueness of this face perception process. The special nature of faces has not been lost on roboticists, who often focus extensively on how to design non-verbal cues derived from the face region (e.g., facial expressions) in order to be easily understandable by humans or to avoid negative consequences associated with robot faces that are of ambiguous human-likeness (i.e., uncanny valley; Mathur and Reichling, 2016).

However, robots challenge both the familiarity and motivational components of face perception. In terms of the familiarity component of face perception, because robot are not (yet) part of everyday life, human face recognition areas did not evolve to be proficient at detecting robot faces, as demonstrated by poor performance at distinguishing similar looking robots. Furthermore, because robot faces do not contain the same familiar features as human faces (e.g., lack of eyebrows) and often do not display human-typical spatial relationships between facial features (Blow et al., 2006), it is possible that their faces do not fully engage face-typical processing. However, research suggests that non-face stimuli can be processed like faces when sufficient familiarity has been generated through perceptual learning (Nussbaum, 1999; Tanaka, 2001; Curby and Gauthier, 2009). Dog experts, for example, show an inversion effect (i.e., face-like processing) when viewing dogs—an effect not observed for non-experts.

In terms of the motivational component of face perception, robots' lack of apparent humanness (Kuchenbrandt et al., 2013) undermines motivation to encode them, making it less likely that they are individuated. This is in line with past research showing that perceivers often fail to individuate even human targets that do not belong to their own social group (Bernstein et al., 2007). Further, given that robots are not actually human, past research has shown that excluding others from the human ingroup (i.e., dehumanization) makes them being seen as interchangeable (Nussbaum, 1999). One way to increase motivation to discriminate others is making them seem similar to the perceiver, thereby enhancing their chances of being perceived as ingroup (Haslam and Loughnan, 2014), or increasing their motivational relevance in other ways. For example, making perceivers outcome dependent on targets (Young and Hugenberg, 2012) or explicitly rewarding discrimination can improve perceivers' ability to distinguish between them (Hugenberg et al., 2007) and can improve recognition and facilitate perceivers building further familiarity. Notably, there is evidence that similar mechanisms also affect how non-human agents are perceived. Almaraz et al. (2018), for instance, demonstrated that leading perceivers to believe that novel non-human agents had humanlike capacities made it easier for perceivers to discriminate between them. Analogously, research in human–robot interaction (HRI) has shown that robots can be made perceived as ingroup members by displaying physical (i.e., gender; Kuchenbrandt et al., 2011, 2013) or behavioral signs (i.e., mannerisms; Bartneck et al., 2007; Oistad et al., 2016) of human-likeness, suggesting that the degree to which a robot is perceived as human-like could potentially improve discrimination via increasing an observer's motivation to do so.

## Aim of study

Here, we investigate whether social robots' faces are sufficiently human-like to trigger face-typical processing. Given that perceivers typically have little familiarity with robots, and may be less motivated to individuate robot faces, we hypothesized that robot faces would be unlikely to receive face-like configural processing. We operationalized face-like processing using the face inversion paradigm that is commonly employed in studies of human face encoding. In this paradigm, participants first complete a learning phase, in which faces are presented to participants in upright position and participants are asked to memorize them; during the subsequent recognition phase, both previously seen and new face stimuli are presented, and participants indicate whether they had seen each face before. Most importantly, during the recognition phase, some of the faces are presented upright and some upside down and discrimination performance for upright vs. upside down stimuli is compared using the Signal Detection index sensitivity (*d*′). For human faces, presenting a previously encountered face stimulus upside-down reduces recognition performance due to a disruption of face-typical processing (i.e., inversion effect). For non-face stimuli, the inversion effect is typically absent (Yin, 1969). If robot faces are processed differently from human faces, the inversion effect should be reduced compared to human faces, indicating attenuated face-typical processing.

## Methods and materials

### Participants

A power analysis conducted with the effect size (η^2^ = 0.07) of a previous experiment employing the inversion task in a similar within-subjects design (Young et al., 2014; Experiment 2), indicated a sample size of *n* = 86 would give us more than a 95% to obtain significance, given an effect. To compensate for participants we would likely remove due to poor performance, we oversampled, collecting 104 participants (*M* = 37.25, range: 20–72, 59 females). Of these, 13 participants were removed due to performing below chance on the inversion task, leaving 91 participants. Participants were recruited via Amazon's Mechanical Turk and participated in the experiment in exchange for compensation. All participants provided informed consent, and the research was approved by the Office of Research Integrity and Assurance at the University.

### Apparatus

The experiment was run on the Inquisit 5 (2016) platform online. Inquisit allows collection of behavioral data remotely over the web via participant keystrokes. Participants downloaded the software and participated in the experiment locally on their computer. Screen size, keyboard, and refresh rate depended on the settings of the participant's individual computers and were not controlled.

### Stimuli

The sample of faces consisted of 40 robot faces, and 40 white male human faces. Robot face stimuli were collected by first compiling images of robots via the ABOT database (Anthropomorphic robot database; Phillips et al., 2018) and internet searches with the only inclusion criteria being that faces contained eyes. Our rationale for limiting inclusion criteria to eyes, without including other features (i.e., eyebrows, eyes, nose, mouth) is that prior face perception research indicates eyes are the defining feature of face-likeness (Looser and Wheatley, 2010). Additionally, given that many robots only contain eyes, this inclusion criteria allowed us to include robots with a range of features typical in social robots, increasing the generalizability of our findings. Furthermore, exclusion criteria was limited to android robots, who bear such strong resemblance to human faces, prior research indicates participants may confuse them for human stimuli at short stimulus times (Wheatley et al., 2011).

**Figure 1:**
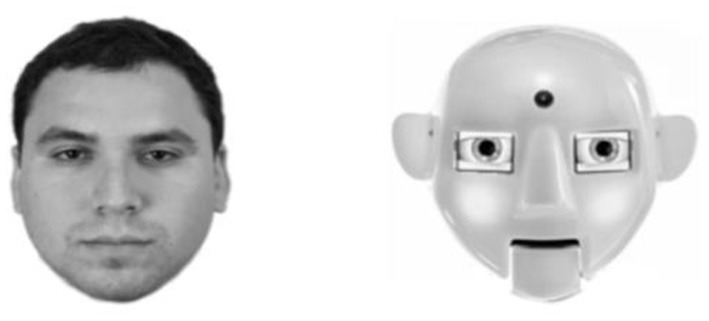
Example of human **(left)** and robot **(right)** stimulus.

**Figure 2:**
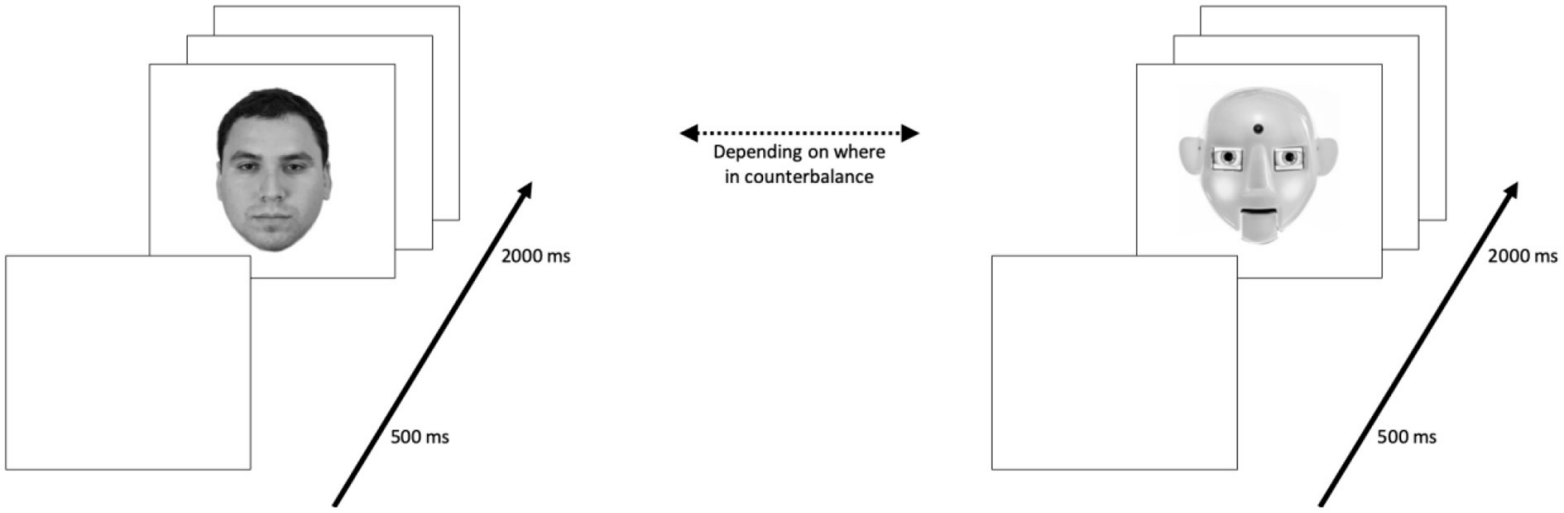
Learning phase. Participants were first instructed they would see 20 upright human (or robot depending on counterbalance) faces and should attend to these faces in order to recognize them later. Participants then proceeded to the learning phase in which they passively viewed the 20 target faces which were displayed in a randomized order for 2,000 ms each, with an ITI of 500 ms.

After a robot face was identified, we searched for images of them displayed in frontal aspect and which the entirety of the face was visible from the top of the head to the bottom of the chin. Afterwards, robot bodies and peripheral background areas were then cropped so that only the robot's face remained. Then, the robot torso and all peripheral information were cropped from the image and Human face stimuli were obtained from the Chicago Face Database (Ma et al., 2015). All faces were then converted to gray scale and presented on a white background that measured 768 × 768 pixels; see Figure 1 for example human and robot stimuli and Figure 2 for all robot stimuli.

### Task

The inversion task consisted of a learning phase and a recognition phase. During the learning phase, participants passively viewed 20 target faces displayed in a randomized order for 2,000 ms each, with an inter-trial interval (ITI) of 500 ms. All faces had an equal chance of being displayed or not displayed; see Figure 2.

**Figure 3:**
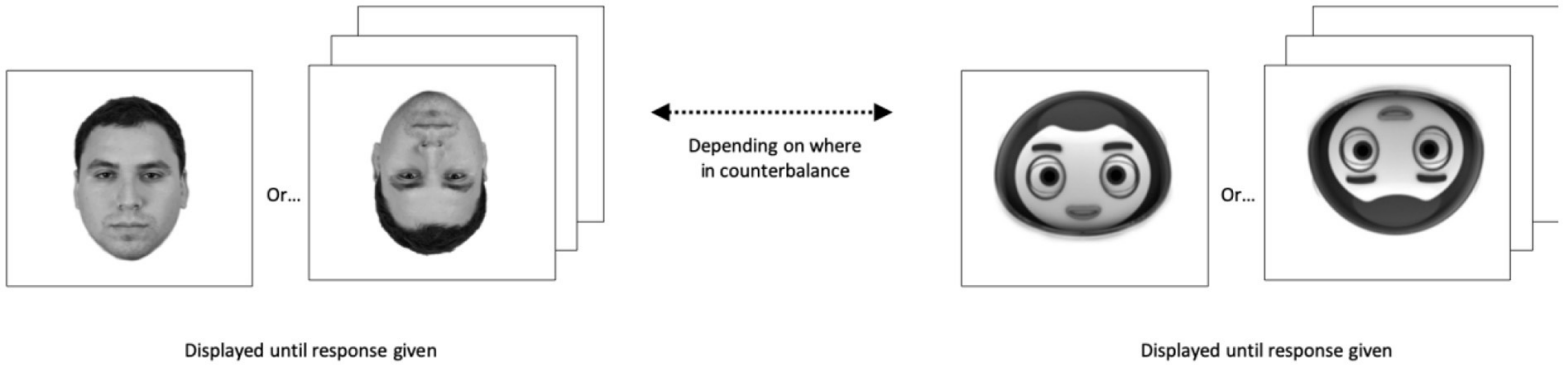
Recognition phase. Participants were instructed they would see another series of human (or robot depending on counterbalance) faces, some of which they had already seen and had some of which they had not. They were also told some of the faces they had not and had not seen would be presented upside-down and that their task was to report whether or not they had seen the face during the learning task.

During the recognition phase, the faces from the learning phase were presented with 20 new faces of the same agent type. Additionally, half of the original and half of the new faces were inverted, enabling us to gauge the impact of disrupting configural processing. For each trial of the recognition phase, participants had to indicate whether they had seen a given face during the previous learning phase by pressing either the “D” key if they had seen the face, and the “K” key if not. A trial did not end until participants gave a response. All distractor and target faces had an equal chance of being displayed upright or inverted; see Figure 3.

### Procedure

Participants first provided informed consent and then received instructions explaining the experimental procedure. Specifically, they were told that during the experiment they would first be asked to memorize a subset of the faces before being asked to recognize those faces when being presented together with a set of new faces.

Participants were then directed to the inversion task, which consisted of a learning and a recognition phase blocked by agent condition (human or robot), such that participants performed both the learning and recognition phase for one agent first (e.g., human) before completing both sequences for the other agent (e.g., robot). The order in which participants performed the task with a particular agent was randomized across participants.

At the beginning of the learning phase, participants were instructed that they would see 20 upright human or robot faces (depending on the current agent condition) onscreen and should attend to these faces in order to recognize them later. After successful completion of the learning phase, the participants proceeded to the recognition phase. During the recognition phase, they were instructed that they would see another series of faces, some of which they had already seen during the learning phase, and some of which they had not. Additionally, they were told that their task was to respond as quickly and accurately as possible as to whether they had seen the presented face during the learning phase, by pressing the “D” key, or not, by pressing the “K” key. Once the learning a recognition phase was completed for the first agent (e.g., human), participants completed the same sequence for the other agent (e.g., robot). Finally, participants were thanked for their participation, received their compensation, and were debriefed.

## Analysis

To assess face recognition performance, we first calculated hit rates (familiar face correctly identified), misses (familiar face not correctly identified), correct rejections (unfamiliar face correctly rejected), and false alarms (unfamiliar face falsely identified) during the recognition phase and used them to calculate accuracy within the Signal Detection Framework. Within the Signal Detection Framework, traditionally, hit rates are calculated by dividing the number of hits by the sum of hits and misses. False alarm rates are calculated by dividing false alarms by the sum of false alarms and correct rejections. Hit rate and false alarms are then z-transformed and subtracted from each other, for human and robot faces separately, and used to calculate sensitivity (*d*′) scores—a measure of target detection that accounts for both hit rates and false alarms (Stanislaw and Todorov, 1999). Since participants who had 100% hit-rates or 0% false alarm rates would result in a *d*′ score that is infinite, a log-linear approach was used to adjust *d*′ scores (see Hautus, 1995; Stanislaw and Todorov, 1999; for detailed procedure). The data from 13 participants were excluded whose performance was worse than chance (*d*′ = 0).

For the remaining participants, *d*′ scores were entered into a 2 (*Agent*: human vs. robot) × 2 (*Orientation*: upright vs. inverted) repeated-measures ANOVA. If configural processing was attenuated, a significant interaction effect between agent and orientation would be expected, such that the difference in *d*′ between the upright and inverted face presentation condition would be more pronounced for the human face stimuli than the robot face stimuli.

## Results

The ANOVA showed a main effect for *Orientation*, such that participants showed a better recognition performance for upright (*M* = 1.36, *SD* = 0.83) compared to inverted (*M* = 0.90, *SD* = 0.81) faces; *F*_(1, 90)_ = 56.75, *p* < 0.001. Additionally, a significant main effect of *Agent* showed that participants had better recognition performance for robot (*M* = 1.23, *SD* = 0.86) compared to human faces [*M* = 1.04, *SD* = 0.84; *F*_(1, 90)_ = 6.46, *p* = 0.013].

**Figure 4:**
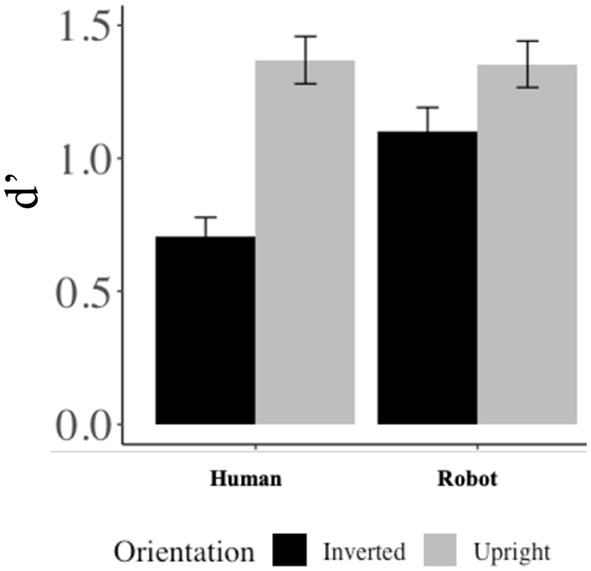
Results. For human faces, there was a larger difference in recognition performance when comparing faces presented upright and inverted than for robots.

Most importantly, and in line with our hypothesis, the interaction effect between *Agent* and *Orientation* was also significant, such that there was a larger difference in recognition performance for human faces that were presented upright (*M* = 1.37, *SD* = 0.85) vs. upside-down (*M* = 0.71, *SD* = 0.69) than for robot faces [upright: *M* = 1.35, *SD* = 0.83; inverted: *M* = 1.10, *SD* = 0.87; *F*_(1, 90)_ = 8.93, *p* = 0.004]. *Post-hoc t*-tests indicated a significant difference in upright vs. upside-down recognition performance (i.e., inversion effects) for both human [upright: *M* = 1.37, *SD* = 0.84; inverted: *M* = 0.71, *SD* = 0.69; *t*_(90)_ = 7.08, *p* < 0.001] and robot faces [upright: *M* = 1.35, *SD* = 0.83; inverted: *M* = 1.10, *SD* = 0.87; *t*_(90)_ = 2.83, *p* = 0.006]; see left-hand side of Figure 4.

## General discussion

The goal of the current experiment was to examine whether faces of current social robots were sufficiently human-like to elicit face-typical processing. Comparable to previous studies on human face perception, face-typical processing was assessed via the face inversion paradigm, which shows a significant decrease in recognition performance of previously encountered faces if those faces were presented upside down vs. upright (i.e., inversion effect).

In line with predictions, we find that robot faces elicit less face-like processing than do human faces. If robot faces engaged face-typical mechanisms less strongly than human faces, human operators would be expected to have difficulties distinguishing robot agents in mixed human-robot teams, with potentially negative consequences for trust calibration and allocation of task resources.

## Theoretical contributions

The findings indicate that although robot faces possess some human-like features, they do not engage face-typical processing to a similar extent as human faces. In light of previous studies that show reduced inversion effects for non-social object stimuli like houses compared to human face stimuli (Yin, 1969), the current findings suggest that robot stimuli may cause under-activation in social brain areas such as the FFA and STS, causing them to be processed more like objects than faces, which might not only have negative consequences on face-related processes like individuation but also impact the degree to which robots are perceived as social entities. This can have severe consequences for HRI: a reduced ability to distinguish robot team members can lead to problems with trust calibration and resource allocation (Keller and Rice, 2009; Geels-Blair et al., 2013) and a reduced perception of robots as social entities can lead to attenuated social-cognitive processing on joint human-robot tasks (see Wiese et al., 2017 for a review). Both issues are discussed in further detail below.

The fact that robots are not processed in a face-like manner indicates that perceivers may lack the familiarity and/or the motivation to engage social brain areas with robot stimuli. This may not necessarily lead to an inability to discriminate robot stimuli by default (as is indicated in an overall good recognition performance for robot faces) but it indicates that robot faces are processed more like objects (Yin, 1969) rather than unique social agents with indentifiable personality attributes. Indeed, although perceptual discrimination *per se* does not necessarily need face-typical processing but could also be accomplished via object-like processing, social perceived stimuli are discriminated in terms of their perceptual features and accompanying identifiable personality characteristics and attributes. A similar effect has been observed in human face perception, such that faces of members of social outgroups (e.g., racial outgroups) engage brain areas more associated with object-like processing rather than social processing afforded to faces of members of social ingroups (e.g., same racial group as observer). In particular, the Occipital Gyrus (Gauthier et al., 1999) is associated with processing objects and is linked to categorical thinking, the activation of stereotypes (Hugenberg et al., 2010), and overall failures to see targets as having sophisticated humanlike faculties (Cassidy et al., 2017). Of particular importance, object-like perception could reduce the degree to which robots are perceived as agents with sophisticated processing capacities (i.e., mind perception, Wiese et al., 2017). On the other hand, it has been shown that processing non-human faces in a configural manner is linked to seeing those agents as having sophisticated minds (Young et al., 2019). Given that mind perception is a pre-requisite for engaging social-cognitive processes, robots that fail to trigger face-typical processing could also be accompanied by impairments of higher-order social mechanisms like theory of mind (see Baron-Cohen, 2000 for a review). In terms of future research, it will be important to examine to which extent building expertise and familiarity with robots will help shift perception from (non-social) object-like to (social) face-like processing.

Finally, object-like processing may also reduce people's motivation to engage with robot stimuli in everyday interactions and reduce their willingness to apply human-like social scripts and norms to the interaction. It could also significantly impact discrimination performance *in vivo*, such that people may pay less attention to robot than human faces in realistic interactions (rather than paradigms where they are instructed to pay attention to the robot faces), which may negatively impact stimulus discrimination (independent of whether it is based on object- or face-like processing). Perceiving robots as objects potentially also reduces the extent to which they are perceived as belonging to the “human” ingroup, which may reduce the motivation to pay attention to individuating agent features even more.

## Practical contributions

This study also has important practical implications for HRI. First, not being able to distinguish robot team members that have different reliabilities and capabilities leads to miscalibrated levels of trust such that we may distrust skilled robots and over rely on less skilled ones. Robots with human-like features may be more likely to be processed as unique social, rather than homogenous, entities based on our life-long experience with human faces, and may motivate people to see robots as individuals when they display human-like face features that activate social brain areas (see Haxby et al., 2000 for a review). Previous research in psychology has identified several low-level perceptual features that are unique to human faces and as such have a high chance of triggering face-typical processing. One such feature is the facial-width-to-height ratio (FWHR), such that faces with low FWHRs (narrower than wide faces) are more likely to be perceived as human-like than faces with high FWHRs (wider than narrow faces; Deska et al., 2018). Upon visual inspection, the robot faces used in the current research were oftentimes round and more symmetric in terms of FWHR than human faces, which may have impacted the degree to which they triggered face perception. However, because the robot stimuli were based on an internet search using pre-defined criteria that did not include FWHR (see section Methods), this should be taken as a reasonably representative sample of facial features of current robots rather than a biased selection of stimuli. To examine the effect of FWHR on face perception in robots further, future studies should experimentally manipulate the FWHR of robot faces (i.e., human-like vs. non-human-like FWHR) and measure the effects of such a manipulation on the face inversion effect.

Another hallmark human face-likeness is that certain features need to be present, namely: eyes, eyebrows, mouth, and nose. A lack of one or more features is associated with a reduction in human-likeness (Deska and Hugenberg, 2017). In line with this, Looser and Wheatley (2010) showed that when face morphs varied in human-likeness from doll to human, as the amount of human facial features increased and doll features decreased, the facial stimuli were perceived as more human-like. The authors also provide evidence that the most important facial feature conveying human-likeness is the presence of eyes (Looser and Wheatley, 2010), while other authors showed that eyes that deviate from human size and/or shape are associated with violations of human-likeness and the uncanny valley phenomenon (Kätsyri et al., 2015). On the other hand, the presence of human-like eyes has been shown to increase the level of anthropomorphism that is induced by very non-human stimuli like light switches, cars, or geometric shapes (Aggarwal and McGill, 2007; Gao et al., 2010; Ahn et al., 2014). In addition to the mere presence of human facial features, the spatial relationship between the features also plays a role for configural processing (Maurer et al., 2002). For instance, placing eyes at unusual locations in human faces disrupts face processing and reduces the inversion effect (Maurer et al., 2002). Since most current robots do not contain all essential human facial features or display facial features in an exaggerated fashion that does not follow human-typical spatial relationships, future research needs to examine to what extent these design features affect face-typical processing in robots.

Lastly, the current research also highlights the suitability of traditional psychological paradigms, such as the face inversion effect, to objectively evaluate the design of social robots and to make specific predictions about how specific design choices will impact social-cognitive mechanisms in HRI. Specifically, because the face inversion effect is an early-stream perceptual effect rather than a late-stage judgment effect, it is likely less susceptible to response biases, allowing for a more direct measure of the extent to which a robot face is being perceived as human-like. Given that past research has reliably linked outgroup membership status to a reduction in face-typical processing, the current research provides evidence that robots are often perceived as lacking key perceptual components of the “human ingroup.” While not being the first to demonstrate the outgroup status of social robots (Eyssel and Loughnan, 2013) or impaired configural processing of robots (Zlotowski and Bartneck, 2013), we are the first ones to show its' consequences for face perception.

## Limitations and future directions

The current study is bounded by certain limitations. First, it is not clear to which degree the current results can be explained by a lack of perceptual experience with the stimuli as opposed to motivational factors related to the relevance of robot stimuli to oneself. As discussed earlier, it is reasonable to assume that both familiarity and motivational factors may explain the observed reduction in face-typical processing for robot faces. However, because the current study cannot answer which of the factors played the major role, future studies need to examine the particular contribution of these two factors to face perception in HRI.

Second, while we argue that discrimination of robot faces may enable better trust calibration and allocation of resources in human–robot teams, the current study does not directly measure how reductions in face-typical processing affects performance measures in human–robot teams. The challenge for future research will be to link attenuated face processing to system-wide trust and interventions designed to improve configural processing to component-specific trust.

Third, it is also important to mention that the external validity of the current research may be limited since images of robot faces were used instead of embodied robot platforms. This could particularly impact participants' motivation to pay sufficient attention to identity-specific features. Furthermore, although objective criteria for image selection were defined prior to conducting the image search, we cannot fully exclude that the observed effect is to a certain degree due to the specific robot images used here.

## Conclusions

The current study uses objective measures to show that prototypical robot faces are perceived as less face-like than human faces and discusses implications for human–robot interaction, as well as potential intervention strategies that could improve face processing of robot stimuli. Because face-like processing can prevent issues in human-robot teaming related to miscalibration of trust, our research has important practical implications for HRI. Future research will explore practical applications further and extend our results to embodied robot platforms.

## Data availability statement

The raw data supporting the conclusions of this article will be made available by the authors, without undue reservation.

## Ethics statement

The studies involving human participants were reviewed and approved by Office of Research Integrity and Assurance at George Mason University. The patients/participants provided their written informed consent to participate in this study.

## Author contributions

AM and EW conceptualized the study. AM collected the data and analyzed the data. KH, EW, and AM interpreted the results and wrote the manuscript. All authors contributed to the article and approved the submitted version.

## Funding

This material is based upon work supported by the Air Force Office of Scientific Research under award number 21USCOR004.

## Conflict of interest

The authors declare that the research was conducted in the absence of any commercial or financial relationships that could be construed as a potential conflict of interest.

## Publisher's note

All claims expressed in this article are solely those of the authors and do not necessarily represent those of their affiliated organizations, or those of the publisher, the editors and the reviewers. Any product that may be evaluated in this article, or claim that may be made by its manufacturer, is not guaranteed or endorsed by the publisher.

## Author disclaimer

The views expressed in this paper are those of the authors and do not reflect those of the U.S. Air Force, Department of Defense, or U.S. Government.
